# Supplementary material for: A Critical Appraisal of the Measurement of Adaptive Social Communication Behaviors in the Behavioral Intervention Context
Source: Behav Sci (Basel). 2025 May 23;15(6):722. doi: 10.3390/bs15060722 (PMC12189064; doi:10.3390/bs15060722)
Supplement: Supplementary file 1 [file behavsci-15-00722-s001.zip › behavsci-3547950-supplementary.pdf]

Table S1. Comparison of SCI-relevant items on adaptive function and autism symptom measures.

SCI Content	Adaptive Function Measure	SCI Item Stem	Autism Symptom Measure	SCI Item Text
Perspective taking	Vineland-3	"Realizes when others are happy, sad,…"	ASDQ	"Seem to understand what others are thinking or feeling"
			SRS	"…able to understand the meaning of other people's tone of voice…"
Response to name	Vineland-3	"Looks when someone calls...name"	ASDQ	"Respond quickly when their name is called? *(Examples: looking at the person who called their name, acknowledging the other person)"*
Appropriate response to others	Vineland-3	"Smiles when…gets praise…"	ASDQ	"Respond appropriately when others approach them? *(Examples: smiling, nodding, saying something back)"*
			SCQ	"Smile back if someone smiles"
Imitation of actions and facial expressions	Vineland-3	"Imitates…when…make a happy, sad,…face"	ASDQ	"Imitate mannerisms and actions by others?"
			SCQ	"Spontaneously copy you or what you are doing…"
			SRS	"…imitate others' actions"
Eye contact	Vineland-3	"Makes good eye contact…"	ASDQ	"Make expected eye contact? *(not too brief, too intense, or looking past people)"*
			SCQ	"…usually look at you directly in the face…"
			SRS	"Avoids eye contact…"

Interest in peers	Vineland-3	“Acts interested in children…”	ASDQ	“Seek out playful interactions, playmates, or friendships?”
			SCQ	“…interested in other children…same age…”
Offering comfort to others	Vineland-3	“Uses actions or words to show…concerned about them”	ASDQ	“Offer comfort to others when they are upset or sick?”
			SRS	“Offers comfort to others…”

Note. Only items stems are provided for Vineland-3, ABAS-3, SRS, and SCQ as these measures are proprietary and copyrighted.

Figure S1. Flow diagram for the initial referral and CIBI outcome assessment process.

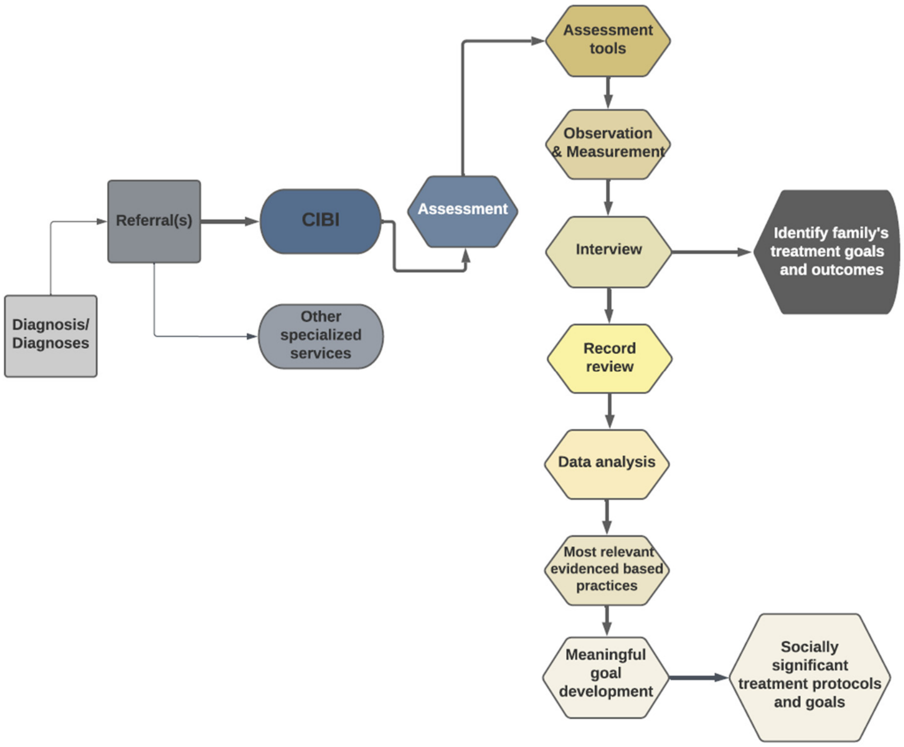

Note. CIBI = comprehensive and intensive behavioral intervention

Supplemental Background. Session-based and fidelity and quality CIBI assessments.

Session-based intervention target data collection, has been a staple of the behavioral intervention process since its inception. CIBI session data are typically collected on a trial-by-trial, event-by-event, or across a segment of time and are displayed and analyzed to inform treatment protocol modifications, changing specific intervention targets, or moving from acquisition to mastery to generalization and maintenance (Johnston et al., 2020). As such, and due to the highly individualized and detailed nature of this type of assessment, ongoing CIBI intervention target data collection is necessary for in-the-moment intervention implementation and has some utility for ipsative interpretation (e.g., how many specific behaviors has this individual demonstrated mastery or maintenance). However, intervention target data collection is not able to provide broader evidence regarding the individual's progress relative to normative expectation and ipsative interpretations are unstandardized and highly dependent on the therapeutic context and implementation.

Collection of intervention quality and fidelity data, has received growing attention with digital data collection and session tracking systems permitting the development of novel measures and tools. At present, collection of intervention quality and fidelity data often involves disparate activities such as examining the fidelity of a new therapist (e.g., behavior technician) in implementing a set of behavioral intervention procedures relative to an expert or highly-trained therapist (e.g., appropriate stimulus presentation, prompting, and reinforcement provision) and accurate collection of the above described trial-by-trial, event-by-event, or time segment data. This category can also include collection of a range of other session or episode of care metrics, such as percentage of total services used by the patient, frequency of parent/caregiver involvement, fidelity of implementation, learning opportunities presented to the patient, and many others. Practice guidelines recommend the collection of fidelity data but requirements

again are flexible and dependent on the specific CIBI application. Industry guidelines encourage practitioners to consistently collect treatment fidelity data as well as periodically assess the reliability of data collection methods (Council of Autism Service Providers, 2024), but requirements tend to be flexible and dependent on the specific CIBI application. As with ongoing intervention target data collection, assessment data regarding intervention quality and fidelity add value to understanding and managing CIBI delivery, but have less utility for ascertaining the value of CIBI services to the individual recipient. Put another way, quality and fidelity assessments tend to be more oriented towards the provider or service-line level of analysis, rather than at the level of the individual receiving treatment.

Figure S2. Visual depiction of the application of initial and periodic CIBI outcome assessments to facilitate treatment planning and ongoing clinical management.

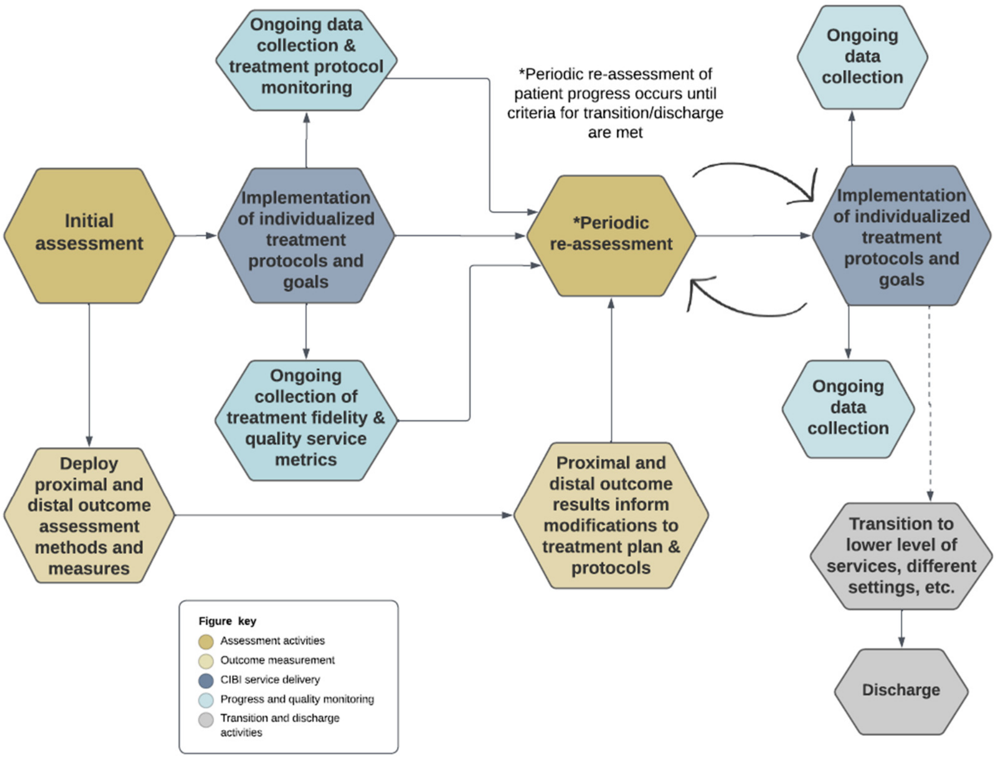

Supplemental Text 1. Roles for CIBI Periodic Outcome and Treatment Planning Assessment.

Developing the initial CIBI intervention strategy. Baseline CIBI outcome assessment, prior to beginning treatment, is key to identifying the initial therapeutic strategy (i.e., setting, group vs. individual services, parent/caregiver training approach, mixture of specific intervention techniques, and direct intervention dosage). Specifically, recent meta-analytic findings indicate that child characteristics are important predictors of intervention outcome, including general cognitive ability or IQ, language, autism symptom level, and adaptive function (Chetcuti et al., under review). At minimum, this suggests that these measures can inform expected rates of progress and developmental trajectory and could drive clinician and caregiver decision-making when shaping the short- and long-term CIBI plan. As mentioned above, while most studies suggest that ABA and NDBI teaching approaches have similar overall efficacy, it is clear that therapists are modifying their implementation of these approaches depending on their appraisal of child's characteristic and needs (Rogers et al., 2021). Indeed, younger individuals and those with less favorable cognitive profiles and/or higher support needs often receive more structured teaching methods and those with more favorable cognitive and behavioral profiles often benefit from more naturalistic teaching procedures. Further, research suggests sequencing distinct treatment approaches may maximize intervention response in children with ASD with minimal speech (Kasari et al., 2014). It is also clear, that the level of intensity of intervention often needs to be titrated depending on the specific characteristics and needs of the patient. This consideration is particularly pertinent given the substantial costs of CIBI. Payors are carefully evaluating treatment authorization requests for justification of medical necessity and intervention intensity. Thus, baseline CIBI assessment may have value for informing intensity level (Frazier et al., in press), expected duration of intervention (Frazier et al., 2021), and specific intervention

methods and sequences (Kasari et al., 2018), including engagement in parent/caregiver training and caregiver-mediated intervention (Minjarez et al., 2024). Through their predictive value, adaptive function measures, at minimum, could play an important part in shaping the initial strategy and identifying key intervention methods (e.g., chaining for teaching practical self-care skills vs. naturalistic reinforcement for building peer relationship behaviors).

Identifying target cognitive and behavioral domains and sub-domains. Instruments within the outcome assessment battery should be chosen to cover key domains relevant to CIBI for autistic people. This includes coverage of developmentally- and diagnostically-relevant sub-domains crucial for more adequately characterizing the patient's strengths and challenges. Many children with ASD have co-occurring mental health conditions (Lai et al., 2019), such as anxiety, ADHD, sleep problems, and mood challenges. Outside of frank DSM-5 diagnoses, many individuals with ASD also show executive functioning/self-regulation challenges as well as severe, disruptive behaviors (Frazier et al., 2022; Lecavalier, 2006). Thus, a CIBI outcome assessment battery with utility for both intervention planning and progress monitoring should include key sub-dimensions of these areas, including hyperactivity, impulsivity, initiation, risk taking, emotion (dys)regulation, irritability, and many others. Furthermore, behavioral challenges in ASD have significant impact on quality of life for the patient and their surrounding environment (Shepherd et al., 2024). Thus, a thorough CIBI battery should include measures beyond core ASD symptoms and adaptive functioning, with particular emphasis on common co-occurring conditions and symptom dimensions and quality of life or well-being in the patient and family. This reinforces the need for efficiency of measurement within each domain, including SCI behavior, to ensure adequate coverage of the broad range of domains relevant to CIBI.

Without broad measurement across at least a subset of these areas, it becomes very difficult to understand and monitor treatment progress.

Recommending specific behaviors for therapist selection. A CIBI outcome assessment battery should include sufficient content coverage to permit the clinician to use results to identify potential behavioral targets (or at least classes of behavior) for goal and objective generation. This requires one of two instrument types: a) a very long instrument that has strong content and construct (sub-domain) coverage and with sufficient items and associated behavioral exemplars to fully and independently inform the intervention planning process OR b) the use of briefer assessments with good measurement precision and domain/sub-domain coverage with results being linked to decision-support intervention target banks based on the individual's scores and response patterns to inform the intervention planning process (Youngstrom et al., 2017). Unfortunately, adaptive function measures tend to fall in an uncanny valley between these two options, with hundreds of items entailing significant resource allocation for clinicians (if an interview version is chosen) or substantial burden to informants (if a caregiver/parent version is chosen) yet lacking sufficient content and social sub-domain coverage of all relevant areas to completely inform intervention target selection.

Monitoring intervention progress. Optimal monitoring of progress during CIBI intervention requires instruments with all of the characteristics described above as well as a sufficiently dynamic score range and conditional reliability across that range to enable detection of change (Jabrayilov et al., 2016). Investigations of CIBI models have shown that even constructs with very strong stability, such as general cognitive ability, adaptive function, or autism symptom level, can show score improvements (Whitehouse et al., 2021). It is crucial though that outcome measures provide scoring approaches that maximize sensitivity to change,

including the provision of item-response theory or Rasch-based theta scores, often translated as growth scale values or growth scores. Demographically-adjusted standard scores are less useful for change measurement during CIBI because they focus on change relative to an (often rapidly moving) normative standard. Growth scores permit evaluation of the individual's progress in more absolute terms without normative adjustment and have the added property of equal-interval measurement ensuring that score changes represent a similar magnitude of change regardless of the initial score level (Farmer et al., 2020). Maximizing sensitivity to change of adaptive functioning measures is particularly important given their strong relationship with age and the fact that many of the replacement behavior strategies adopted in CIBI involve either SCI behaviors or practical living skills.

Highlighting possible intervention modifications. As an individual episode of CIBI delivery unfolds, it is nearly always the case that the intervention plan will require modification, including change in therapeutic intensity or focus, alteration of the therapeutic setting, and service transition or discharge. The need for modification can occur less frequently for individuals who show slower progress in mastering, generalizing, and maintaining skills and for those whom initial target selection approximated their needs and proximal zone of development. In contrast, the need for modification can occur very rapidly for individuals who master, generalize, and maintain skills quickly or for those individuals where initial target selection did not adequately match their developmental needs. Adaptive functioning measures can play an important part in identifying the need for and type of modification. For example, a patient showing substantial progress in mastery of self-care skills at 12-month intervention follow-up may signal the ability to fade or downplay targeting of this sub-domain and/or focus on generalization and maintenance of these skills. The utility of adaptive functioning measures in

fulfilling this role and providing information about potential intervention modification is highly dependent on instrument structure, construct coverage, and sensitivity to change.

Table S2. Specific SCI dimensions identified through factor analytic studies.

**Subdomain**	**Definition**	**Potential additional subdomains/facets**
Social motivation	Drive or desire to interact socially and affiliate with others, independent of the quality of the interaction or overture	Orienting, seeking, liking maintaining
Basic social communication behaviors	Verbal and non-verbal behaviors involved in initiating social interactions and responding to non-social bids	Facial and non-facial communication
Relationship-focused and reciprocal interaction behaviors	More complex skills necessary for initiating, maintaining, and ending social interactions that integrate different modalities (e.g., use of both verbal and non-verbal means)	Play
Perspective-taking or theory of mind	The ability to perceive, interpret and respond to relevant (emotional and non-emotional) social signals	Cognitive and emotional theory of mind

Note. The following large sample studies support SCI sub-dimensions (Chetcuti et al., 2024; Frazier & Hardan, 2017; Phillips et al., 2019; Simmons et al., 2024; Uljarevic et al., 2020).

Supplemental Methods. Vineland-3 and ABAS-3 Literature Search.

To conduct a comprehensive overview of the Vineland-3 and ABAS-3 and their application to behavioral intervention, literature searches were conducted using PubMed. For the Vineland-3, search terms included: Vineland AND adaptive AND (psychometric OR reliability OR validity OR development). For the ABAS-3, search terms included: ABAS OR adaptive behavior assessment system AND adaptive AND (psychometric OR reliability OR validity OR development). PubMed search generated 749 results for the Vineland search and 1,822 results for the ABAS search. Titles and abstracts were reviewed relevance and the reference lists for articles with strong relevance were reviewed to identify any missed publications. The Vineland-3 and ABAS-3 technical manuals were also reviewed for relevant information. Where references provided overlapping information, publications with the largest samples or best methodology were selected and cited below, including information from the technical manuals. Only empirical studies that explicitly provided information on specific aspects of psychometric evaluation, including (i) factor analysis, (ii) invariance testing (including measurement invariance and differential item functioning), (iii) reliability (both classical test theory-based reliability [e.g., internal consistency] and item-response theory-based conditional reliability), and (iv) validity, including convergent and discriminant, were included for the full consideration. Out of 2,571 screened studies, 47 specifically examined relevant psychometric information. Of these 7 examined factor structure (5 Vineland, 2 ABAS), 3 measurement invariance (2 Vineland, 1 ABAS), 8 reliability (6 Vineland, 2 ABAS), 35 construct validity (31 Vineland, 4 ABAS), and 5 sensitivity to change/clinically-meaningful change (5 Vineland, 0 ABAS). It is important to note that, if studies that examine Vineland-3 or ABAS-3 patterns in specific clinical groups are included as assessments of construct validity (criterion or known-groups validity) the number of

studies for this area rises dramatically. However, given the reviews conclusion that, using the existing Vineland-3 and ABAS-3 scores, construct validity (convergent and discriminant) is good to excellent would not substantively change. Thus, only studies that provide correlations representing convergent and discriminant validity with other instruments were included in the construct validity counts. Systematic review papers were not included, but, where relevant, the most recently-published test manuals were included in the above counts. Some papers provided evidence in multiple areas.

Supplemental Text 2. Evidence of Vineland-3 Interview Form superiority.

In the authors clinical experience, in multiple autism-relevant research contexts, and in several real-world CIBI applications, the Comprehensive Vineland-3 Interview Form has been recommended or required instead of use of the Parent/Caregiver Survey Form. This recommendation is justified by stating that the Vineland-3 Interview Form produces a more accurate (valid) assessment of adaptive behavior in the ASD population. This review was not able to identify any studies in ASD or in any developmental disability or neurotypical population that specifically demonstrated greater validity or incremental validity of the interview version of the Vineland-3 (or any Vineland version) over the parent/caregiver survey version for measuring real-world adaptive functioning. A thorough review of the Vineland-3 technical manual also did not uncover any evidence for this possibility. This is surprising given the fact that the Vineland-3 development and standardization process involved a number of carefully-conducted studies, including inter-rater and comparison to Vineland-II studies. Furthermore, the only possible evidence of superiority of the interview version over the parent/caregiver form is presented in Chapter 1 of the Vineland-3 manual. The manual states "Goldstein, Smith, Waldrep, and Inderbitzen (1987) concluded that 'the more open-ended nature of the Vineland ABS procedure frequently promotes response elaboration and elicits more information than does the rating scale' (pg. 5)." However, careful review of the Goldstein et. al. (1987) reveals that this quote is referring to the comparison between the Vineland and Scales of Independent Behavior semi-structured interviews. Thus, this quotation is not actually concerning comparability of interview and survey forms and is about comparisons between two different instruments. The Vineland-3 manual goes on to state that "Despite the substantial benefits of the interview method…" but does not provide any evidence of these benefits. Various potential biases in parent/caregiver

rating are provided but no biases in clinician judgment are reviewed. Thus, the currently available evidence for superiority of the Vineland-3 Interview Form over the Vineland-3 Parent/Caregiver Survey Form is lacking.

Without a strong empirical justification, it seems untenable to recommend the interview version over the parent/caregiver survey version. This is key because the resource allocation difference is not trivial in the CIBI context. The interview version will require about ~1 hour of additional clinician time over the caregiver survey version for administration and this will limit the ability of clinicians in this context to collect other information, as the typical insurance authorization is 8-10 hours and clinicians often need to complete a number of other tasks, including direct observation of behavior, skills-based or criterion-referenced assessments, functional behavioral assessments (if any challenging behavior are present), parent interviews for gathering key environmental and social validity information, and other norm-referenced assessments to assess other domains of functioning.

Figure S3. Example continuous norming for total sleep problems using generalized additive modeling with an interaction between age and sex across a wide age range (2 to 90) in a medium-sized normative sample (N=536).

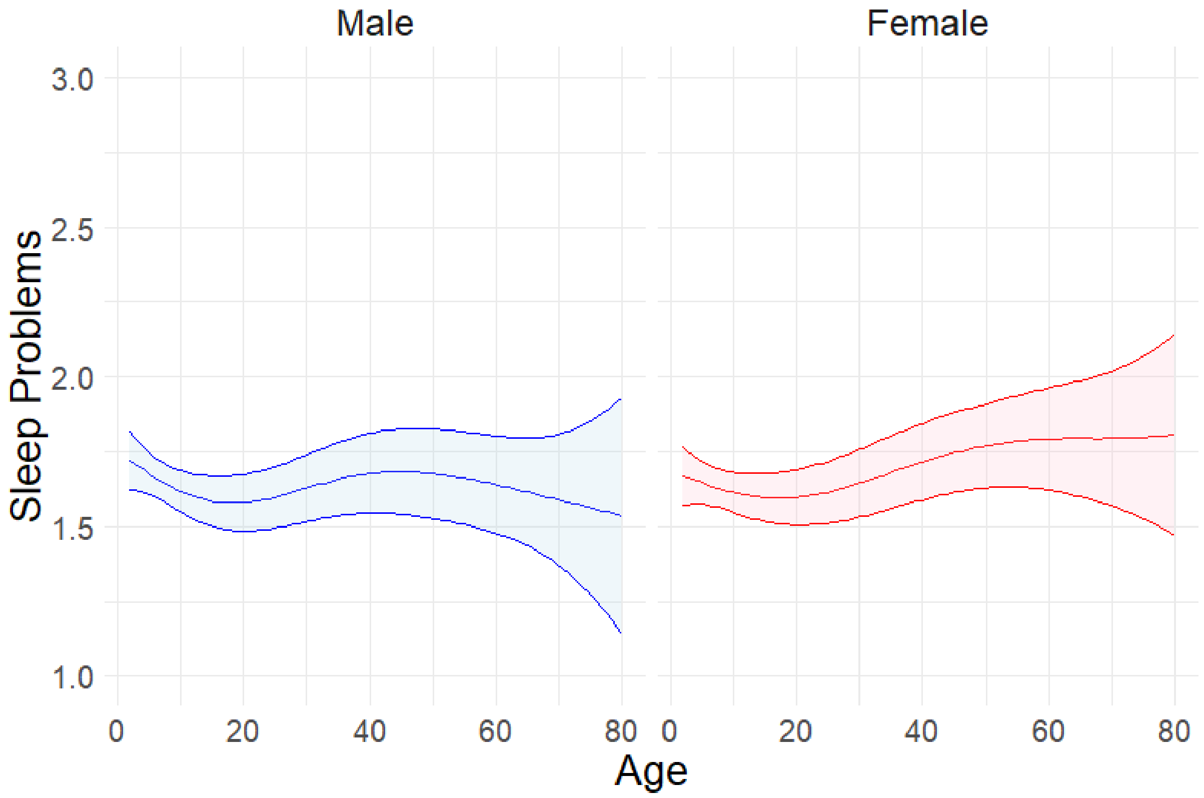

Figure S4. Proposed process for integrating results from briefer standardized, norm-referenced assessments, including measures of adaptive function, with results from skills-based / criterion-referenced assessments to develop a CIBI treatment protocol.

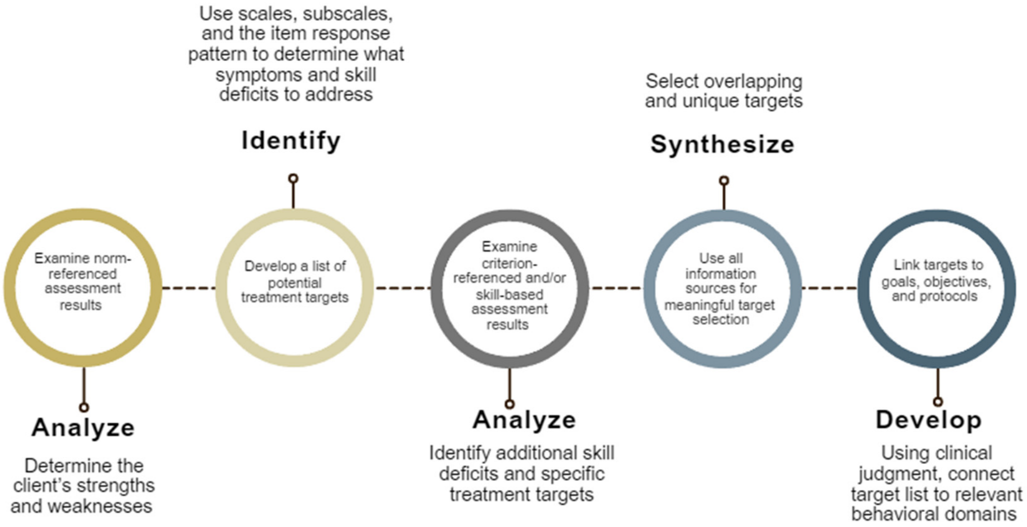

References

Chetcuti, L., Hardan, A. Y., Spackman, E., Frazier, T. W., Youngstrom, E. A., & Uljarevic, M. (2024). Characterizing transdiagnostic processes underlying the drive to socially engage: Multimeasurement factor analytic investigation. *Journal of Child and Adolescent Psychiatry Open*. https://doi.org/https://doi.org/10.1016/j.jaacop.2024.09.007

Chetcuti, L. C., Uljarevic, M., Hardan, A. Y., Gengoux, G. W., Trembath, D., Vadgama, Y., Varcin, K., Vivanti, G., Whitehouse, A. J. O., & Frazier, T. W. (under review). Characterizing Predictors of Response to Behavioral Interventions for Children with Autism Spectrum Disorder: A Meta-Analytic Approach.

Council of Autism Service Providers. (2024). *Applied Behavior Analysis Practice Guidelines for the Treatment of Autism Spectrum Disorder: Guidelines for Healthcare Funders, Regulatory Bodies, Service Providers, and Consumers* (Third Edition ed.) https://assets-002.noviams.com/novi-file-uploads/casp/pdfs-and-documents/ASD_Guidelines/ABA_Practice_Guidelines_3_0.pdf

Farmer, C. A., Kaat, A. J., Thurm, A., Anselm, I., Akshoomoff, N., Bennett, A., Berry, L., Bruchey, A., Barshop, B. A., Berry-Kravis, E., Bianconi, S., Cecil, K. M., Davis, R. J., Ficicioglu, C., Porter, F. D., Wainer, A., Goin-Kochel, R. P., Leonczyk, C., Guthrie, W., . . . Miller, J. S. (2020). Person Ability Scores as an Alternative to Norm-Referenced Scores as Outcome Measures in Studies of Neurodevelopmental Disorders. *Am J Intellect Dev Disabil*, *125*(6), 475-480. https://doi.org/10.1352/1944-7558-125.6.475

Frazier, T. W., Chetcuti, L. C., & Uljarevic, M. (in press). Letter to the Editor: Evidence that Intervention Dosage Does Associate with Better Outcomes in Autism *JAMA Pediatrics*.

Frazier, T. W., Crowley, E., Shih, A., Vasudevan, V., Karpur, A., Uljarevic, M., & Cai, R. Y. (2022). Associations between executive functioning, challenging behavior, and quality of life in children and adolescents with and without neurodevelopmental conditions. *Frontiers in Psychology*. https://doi.org/10.3389/fpsyg.2022.1022700

Frazier, T. W., & Hardan, A. Y. (2017). Equivalence of symptom dimensions in females and males with autism. *Autism*, *21*(6), 749-759. https://doi.org/10.1177/1362361316660066

Frazier, T. W., Klingemier, E. W., Anderson, C. J., Gengoux, G. W., Youngstrom, E. A., & Hardan, A. Y. (2021). A Longitudinal Study of Language Trajectories and Treatment Outcomes of Early Intensive Behavioral Intervention for Autism. *Journal of Autism and Developmental Disorders*, *51*(12), 4534-4550. https://doi.org/10.1007/s10803-021-04900-5

Goldstein, D. J., Smith, K. B., Waldrep, E. L., & Inderbitzen, H. M. (1987). Comparison of the Woodcock-Johnson Scales of Independent Behavior and Vineland Adaptive Behavior Scales in Infant Assessment. *Journal of Psychoeducational Assessment*, *5*, 1-6.

Jabrayilov, R., Emons, W. H. M., & Sijtsma, K. (2016). Comparison of Classical Test Theory and Item Response Theory in Individual Change Assessment. *Appl Psychol Meas*, *40*(8), 559-572. https://doi.org/10.1177/0146621616664046

Johnston, J. M., Pennypacker, H. S., & Green, G. (2020). *Strategies and tactics of behavioral research and practice.* (4th ed.). Routledge/Taylor & Francis Group.

Kasari, C., Kaiser, A., Goods, K., Nietfeld, J., Mathy, P., Landa, R., Murphy, S., & Almirall, D. (2014). Communication interventions for minimally verbal children with autism: a sequential multiple assignment randomized trial. *Journal of the American Academy of*

Child and Adolescent Psychiatry, *53*(6), 635-646. https://doi.org/10.1016/j.jaac.2014.01.019

Kasari, C., Sturm, A., & Shih, W. (2018). SMARTer Approach to Personalizing Intervention for Children With Autism Spectrum Disorder. *Journal of Speech, Language, and Hearing Research*, *61*(11), 2629-2640. https://doi.org/10.1044/2018_JSLHR-L-RSAUT-18-0029

Lai, M. C., Kassee, C., Besney, R., Bonato, S., Hull, L., Mandy, W., Szatmari, P., & Ameis, S. H. (2019). Prevalence of co-occurring mental health diagnoses in the autism population: a systematic review and meta-analysis. *Lancet Psychiatry*, *6*(10), 819-829. https://doi.org/10.1016/S2215-0366(19)30289-5

Lecavalier, L. (2006). Behavioral and emotional problems in young people with pervasive developmental disorders: relative prevalence, effects of subject characteristics, and empirical classification. *Journal of Autism and Developmental Disorders*, *36*(8), 1101-1114. https://doi.org/10.1007/s10803-006-0147-5

Minjarez, M. B., Gengoux, G. W., Paszek, K., Liang, J. A., Ardel, C. M., Hardan, A. Y., & Frazier, T. (2024). Adherence and Opportunity Frequency as Predictors of Communication Outcomes from Pivotal Response Parent Training. *Journal of Autism and Developmental Disorders*. https://doi.org/10.1007/s10803-024-06447-7

Phillips, J. M., Uljarevic, M., Schuck, R. K., Schapp, S., Solomon, E. M., Salzman, E., Allerhand, L., Libove, R. A., Frazier, T. W., & Hardan, A. Y. (2019). Development of the Stanford Social Dimensions Scale: initial validation in autism spectrum disorder and in neurotypicals. *Mol Autism*, *10*, 48. https://doi.org/10.1186/s13229-019-0298-9

Rogers, S. J., Yoder, P., Estes, A., Warren, Z., McEachin, J., Munson, J., Rocha, M., Greenson, J., Wallace, L., Gardner, E., Dawson, G., Sugar, C. A., Hellemann, G., & Whelan, F. (2021). A Multisite Randomized Controlled Trial Comparing the Effects of Intervention Intensity and Intervention Style on Outcomes for Young Children With Autism. *Journal of the American Academy of Child and Adolescent Psychiatry*, *60*(6), 710-722. https://doi.org/10.1016/j.jaac.2020.06.013

Shepherd, D., Buchwald, K., Siegert, R. J., & Vignes, M. (2024). Using network analysis to identify factors influencing the heath-related quality of life of parents caring for an autistic child. *Research in Developmental Disabilities*, *152*, 104808. https://doi.org/10.1016/j.ridd.2024.104808

Simmons, G. L., Corbett, B. A., Lerner, M. D., Wofford, K., & White, S. W. (2024). Social competence in autism: A structural equation modeling approach. *Autism Res*, *17*(4), 761-774. https://doi.org/10.1002/aur.3108

Uljarevic, M., Frazier, T. W., Phillips, J. M., Jo, B., Littlefield, S., & Hardan, A. Y. (2020). Mapping the Research Domain Criteria Social Processes Constructs to the Social Responsiveness Scale. *Journal of the American Academy of Child and Adolescent Psychiatry*, *59*(11), 1252-1263 e1253. https://doi.org/10.1016/j.jaac.2019.07.938

Whitehouse, A. J. O., Varcin, K. J., Pillar, S., Billingham, W., Alvares, G. A., Barbaro, J., Bent, C. A., Blenkley, D., Boutrus, M., Chee, A., Chetcuti, L., Clark, A., Davidson, E., Dimov, S., Dissanayake, C., Doyle, J., Grant, M., Green, C. C., Harrap, M., . . . Hudry, K. (2021). Effect of Preemptive Intervention on Developmental Outcomes Among Infants Showing Early Signs of Autism: A Randomized Clinical Trial of Outcomes to Diagnosis. *JAMA Pediatr*, *175*(11), e213298. https://doi.org/10.1001/jamapediatrics.2021.3298

Youngstrom, E., Van Meter, A., Frazier, T., Hunsley, J., Prinstein, M., Ong, M., & Youngstrom, J. (2017). Evidence-Based Assessment as an integrative model for applying

psychological science to guide the voyage of treatment. *Clinical Psychology: Science and Practice*, *24*(4), 331-363. https://doi.org/doi:10.1111/cpsp.12207
